# The benefit of HER2-targeted therapies on overall survival of patients with metastatic HER2-positive breast cancer – a systematic review

**DOI:** 10.1186/s13058-015-0648-2

**Published:** 2015-11-17

**Authors:** Diogo Mendes, Carlos Alves, Noémia Afonso, Fátima Cardoso, José Luís Passos-Coelho, Luís Costa, Sofia Andrade, Francisco Batel-Marques

**Affiliations:** CHAD – Centre for Health Technology Assessment and Drug Research, AIBILI – Association for Innovation and Biomedical Research on Light and Image, Azinhaga de Santa Comba, Celas, 3000-548 Coimbra, Portugal; Faculty of Pharmacy, University of Coimbra, Azinhaga de Santa Comba, 3000-548 Coimbra, Portugal; Medical Oncology Service, IPO - Portuguese Institute of Oncology Professor Francisco Gentil, Rua Dr. António Bernardino de Almeida, 4200-072 Oporto, Portugal; Breast Cancer Unit, Champalimaud Cancer Center, Avenida De Brasília s/n, 1400-038 Lisbon, Portugal; Medical Oncology Service, Hospital da Luz, Avenida Lusíada, 100, 1500-650 Lisbon, Portugal; Medical Oncology Service, Santa Maria Hospital, Rua de Santa Marta, 1169-024 Lisbon, Portugal; Market Access Department, Roche Pharmaceuticals, Estrada Nacional 249-1, 2720-413 Amadora, Portugal

## Abstract

**Introduction:**

This study aimed at evaluating the overall survival (OS) gain associated with human epidermal growth factor receptor 2 (HER2)-directed therapies in patients with metastatic breast cancer (mBC).

**Methods:**

A bibliographic search was conducted in PubMed and Cochrane databases. Only phase III randomized controlled trials (RCTs) including HER2-positive (HER2+) mBC patients were included in this review. OS was defined as time from randomization until the occurrence of death from any cause. Studies have been grouped according to the line of treatment, i.e., first-line or second-line or beyond.

**Results:**

Nineteen RCTs were eligible for inclusion, of which 12 assessed therapies targeting HER2+ mBC in the first-line setting. OS improved from 20.3 months in the first RCT (standard chemotherapy; Slamon et al. (N Engl J Med 344:783–92, 2001)) evaluating HER2-targeting therapies to 48 months in the study of Swain et al. (Lancet Oncol 14:461–71, 2013), with triple combination of pertuzumab, trastuzumab and docetaxel. Seven RCTs evaluated the OS of HER2-targeting therapies in the second-line setting and beyond. The OS in second-line setting improved from 15.3 months (capecitabine; Cameron et al. (Breast Cancer Res Treat 112:533–43, 2008)) to 30.7 months (trastuzumab emtansine; Verma et al. (N Engl J Med 367:1783–91, 2012)). In the third-line setting, the association of lapatinib and trastuzumab has demonstrated to improve OS to 4.5 months compared with lapatinib alone (14 months vs. 9.5 months; Blackwell et al. (J Clin Oncol 30:2585–92, 2012)).

**Conclusions:**

HER2-directed therapies had an undeniable beneficial impact on the OS of patients with HER2+ mBC. The triple combination of docetaxel, pertuzumab and trastuzumab is associated with a survival extent of more than 4.5 years, compared with a life expectancy of 1.5 years achieved 14 years ago.

**Electronic supplementary material:**

The online version of this article (doi:10.1186/s13058-015-0648-2) contains supplementary material, which is available to authorized users.

## Introduction

Breast cancer (BC) is the second most common cancer worldwide and, by far, the most frequent among women with an estimated 1.67 million new cases diagnosed in 2012 (25 % of all cancers) (Ferlay et al. [6]). BC is the fifth cause of death from cancer overall (522,000 deaths) and it is the most frequent cause of cancer death in women in less developed regions (324,000 deaths, 14.3 % of the total) (Ferlay et al. [6]). In the developed countries, it is the second cause of cancer death (198,000 deaths, 15.4 %), after lung cancer (Ferlay et al. [6]).

In developed countries between 6 and 10 % of women will have metastatic disease when diagnosed with BC (Dawood et al. [7]); in developing countries this percentage can reach 60 %. Depending on initial stage, tumor biology, and type of treatment scheme received, between 30 and 50 % of women with early BC will relapse (Cardoso et al. [8]).

The amplification of the human epidermal growth factor receptor 2 (HER2) is observed in 25 to 30 % of all BCs (Slamon et al. [1]). Patients with BC with overexpression of HER2 have, originally, a poorer prognosis and shorter overall survival (OS) (Tandon et al. [9]; Slamon et al. [10]).

The development of effective HER2-targeted drugs is considered a major breakthrough in BC therapy. Trastuzumab was the first anti-HER2 drug approved for treatment of HER2-positive (HER2+) metastatic BC, either alone or in combination with chemotherapy (Slamon et al. [1]). This anti-HER2 monoclonal antibody was associated with a significantly longer time to disease progression, higher response rate, longer response duration, and improved overall survival (Slamon et al. [1]). During the last decade, HER2-targeted therapeutic approaches continued to evolve with a positive impact on the survival of the women with HER2+ metastatic BC (Dawood et al. [7]).

This study aimed at evaluating the survival gains associated with HER2-targeted therapies in patients with HER2+ metastatic BC.

## Methods

### Data sources and searches

A bibliographic search was conducted in the PubMed and in Cochrane Central Register of Controlled Trials databases (updated October 2015). The search equation comprised terms referring to HER2+ metastatic BC (Additional file 1). No language restrictions were applied. The references lists of systematic reviews were revised in order to identify further studies.

Two reviewers (DM and CA) independently assessed the titles and abstracts of retrieved articles to determine trial inclusion. In case of disagreement, the opinion of a third investigator was sought (BM).

### Study selection

Only phase III randomized controlled trials (RCTs) including patients with HER2+ metastatic BC have been analyzed, irrespective of the treatment administered (i.e., chemotherapy and/or hormone therapy, chemotherapy and/or hormone therapy plus HER2-targeted therapy).

### Data extraction

Data were abstracted in a standardized format by two independent reviewers (DM and CA). The data retrieved from each publication included study name, bibliographic reference, publication year, total number of patients allocated to each treatment arm on the intention-to-treat (ITT) analysis, characterization of the target population, and efficacy outcomes. The primary efficacy outcome was OS (defined as the time from random assignment until death from any cause), while the secondary outcome was progression-free survival (PFS, defined as the time from randomization until objective tumor progression or death) (FDA [11]). We extracted the median duration (in months) for the outcomes OS and PFS, based on the ITT analysis. Where outcome measures were not reported, we contacted the investigators to provide the data.

Studies were divided according to the line of treatment, i.e., first-line or second-line and beyond.

### Data analyses

Data were analyzed using descriptive statistics. Data analysis was performed using Microsoft Excel 2010 (Microsoft Corporation, Redmond, WA, USA).

## Results

**Fig. 1:**
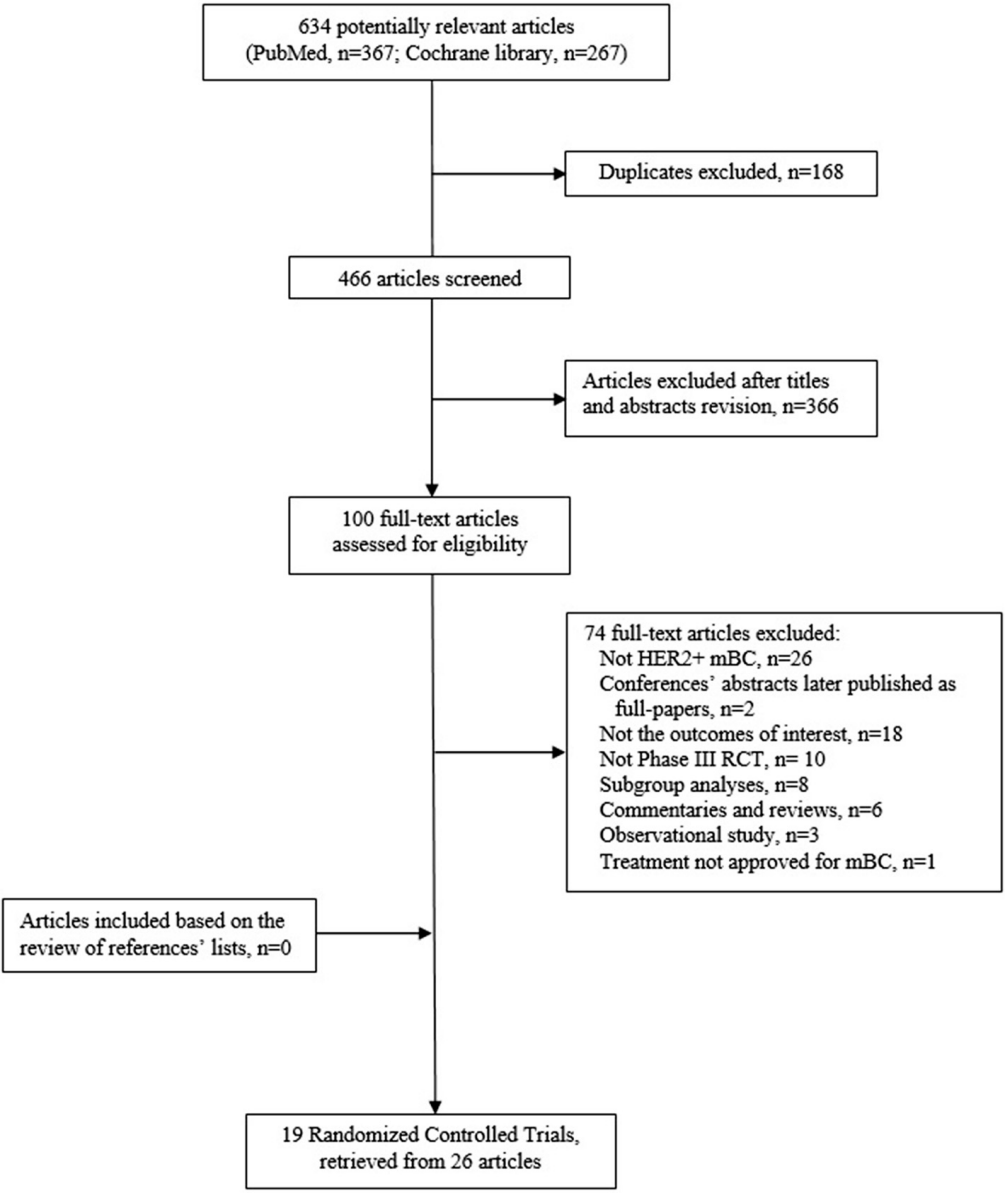
Flow of the search strategy. *HER2+* human epidermal growth factor receptor 2 positive, *mBC* metastatic breast cancer, *RCTs* randomized controlled trials

Figure 1 shows the flow of the search strategy criteria. The electronic databases searches returned 634 potentially relevant articles; 168 references were duplicates. After review of the titles and abstracts, 366 references were refuted and 100 were selected for further evaluation. After the application of the inclusion criteria, 19 RCTs reported in 26 publications were included (Andersson et al. [12]; Baselga et al. [13]; Baselga et al. [14]; Guan et al. [15]; Inoue et al. [16]; Johnston et al. [17]; Kaufman et al. [18]; Robert et al. [19]; Schwartzberg et al. [20]; Slamon et al. [1]; Swain et al. [2]; Swain et al. [21]; Valero et al. [22]; André et al. [23]; Blackwell et al. [24]; Blackwell et al. [5]; Cameron et al. [3]; Cameron et al. [25]; Geyer et al. [26]; Krop et al. [27]; Pivot et al. [28]; Verma et al. [4]; von Minckwitz et al. [29]; von Minckwitz et al. [30]; Gelmon et al. [31]; Hurwitz et al. [32]). Review of published manuscripts’ references did not find any other eligible studies.

### First-line metastatic BC

**Fig. 2:**
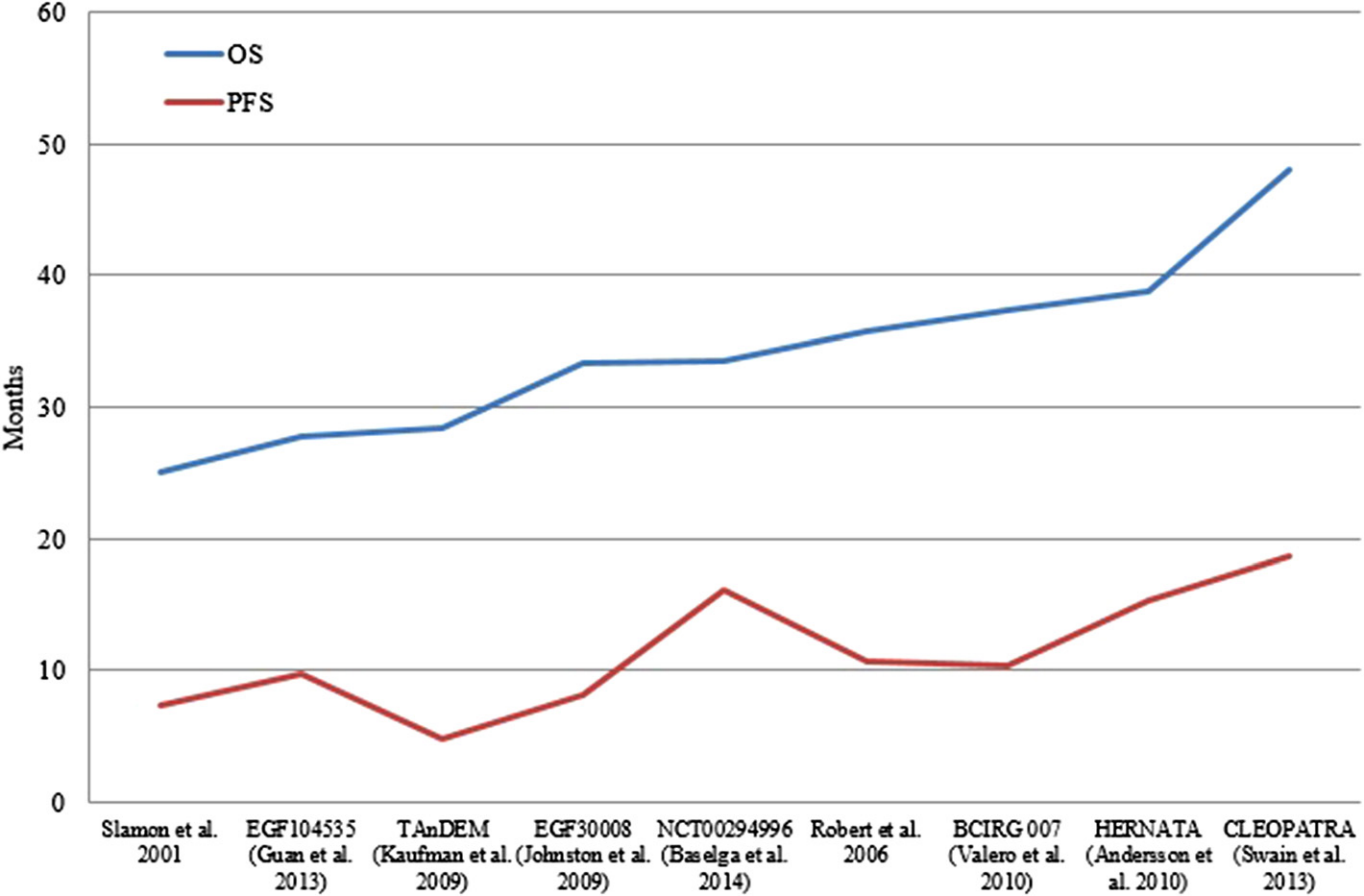
Overall survival (*blue*) and progression-free survival (*red*) of patients diagnosed with metastatic breast cancer receiving first-line treatment, according to the most effective treatment in each study. Results are displayed from the study with lower OS to the study with higher OS. Results from the study by Inoue et al. [16], Gelmon et al. [31], and Hurwitz et al. [32] are not displayed because OS results were not provided. *OS* overall survival, *PFS* progression-free survival

Twelve phase III RCTs assessed therapies targeting HER2+ metastatic BC in the first-line setting (Slamon et al. [1]; Swain et al. [2]; Andersson et al. [12]; Baselga et al. [13]; Baselga et al. [14]; Guan et al. [15]; Inoue et al. [16]; Johnston et al. [17]; Kaufman et al. [18]; Robert et al. [19]; Schwartzberg et al. [20]; Swain et al. [21]; Valero et al. [22]; Gelmon et al. [31]; Hurwitz et al. [32]). The main results of each RCT are shown in Table 1. Figure 2 presents the OS (*blue*) and PFS (*red*) results for patients diagnosed with HER2+ metastatic BC receiving first-line treatment, according to the most effective treatment in each study.

**Table 1:** Summary of design and results of studies assessing first-line therapies for the treatment of patients diagnosed with HER2-positive metastatic breast cancer

Clinical trial	Reference	Year	Target population	T1	T2	Primary endpoint of efficacy	Patients on T1, *n*	Patients on T2, *n*	OS T1	OS T2	HR (95 % CI), *p* value	PFS T1	PFS T2	HR (95 % CI), *p* value
*Chemotherapy ± trastuzumab or lapatinib*														
Slamon 2001	Slamon et al. [1]	2001	Women with progressive mBC that overexpressed HER2 who had not previously received chemotherapy for metastatic disease	Chemotherapy once every 3 weeks for six cycles + trastuzumab loading dose 4 mg/kg on day 1, then 2 mg/kg every week until PD	Chemotherapy alone once every 3 weeks for six cycles	TTP	234	235	25.1	20.3	0.80 (0.64–1.00), *p* = 0.046	6.9	4.5	0.58 (0.47–0.70), *p* < 0.001
JO17360	Inoue et al. [16]	2009	Women with HER2-positive mBC, measurable lesion(s) fulfilling RECIST criteria, ECOG-PS 0–1, and LVEF > 50 %	Trastuzumab loading dose 4 mg/kg then 2 mg/kg every week + docetaxel 60 mg/m2 every 3 weeks	Trastuzumab loading dose 4 mg/kg then 2 mg/kg every week until PD followed by trastuzumab loading dose 4 mg/kg then 2 mg/kg every week + docetaxel 60 mg/m2 every 3 weeks	PFS; OS	53	54	NA	NA	NA	14.6	3.7	4.24 (2.48, 7.24), *p* < 0.01
EGF104535	Guan et al. [15]	2013	Women with newly diagnosed HER2-positive mBC (no prior treatment for metastatic disease was allowed, with the exception of hormonal treatment for patients with hormone receptor–positive disease; prior trastuzumab and/or taxane as neoadjuvant or adjuvant therapy were permitted provided therapy was completed 12 months before study entry)	Lapatinib (1500 mg/d) + paclitaxel (80 mg/m2 once per week for 3 weeks every 4 weeks)	Placebo once per day + paclitaxel (80 mg/m2 once per week for 3 weeks every 4 weeks)	OS	222	221	27.8	20.5	0.74 (0.58, 0.94), *p* = 0.0124	9.7	6.5	0.52 (0.42, 0.64), *p* = 0.001
*Chemotherapy + lapatinib* versus *chemotherapy + trastuzumab*														
MA.31	Gelmon et al. [31]	2015	Women with HER2-positive mBC, ECOG-PS 0–2, no prior therapy with cytotoxics or biologics for recurrent or advanced disease, baseline LVEF ≥ 50 %, measurable or nonmeasurable disease defined by RECIST (v1.0) criteria, and no major end-organ disease	Lapatinib (1250 mg/d) + taxane (paclitaxel 80 mg/m2 once per week on days 1, 8, and 15 of a 28-day schedule or docetaxel 75 mg/m2 once every 3 weeks) for 24 weeks followed by lapatinib (1,500 mg/d) until PD	Trastuzumab + taxane (once per week [4 mg/kg bolus followed by 2 mg/kg maintenance] + once per week paclitaxel; or once every 3 week [8 mg/kg bolus followed by 6 mg/kg maintenance] + docetaxel once every 3 weeks) for 24 weeks followed by trastuzumab (6 mg/kg once every 3 weeks) until PD	PFS	326	326	NA	NA	1.28 (0.95, 1.72), *p* = 0.11	9.0	11.3	1.37 (1.13, 1.65), *p* < 0.001
*Hormone therapy ± trastuzumab or lapatinib*														
TAnDEM	Kaufman et al. [18]	2009	Postmenopausal women with HER2-positive and hormone receptor–positive mBC; LVEF > 50 %; ECOG-PS 0–1; and measurable or evaluable disease; prior chemotherapy for mBC or adjuvant chemotherapy within 6 months was not permitted	Anastrozole 1 mg/day + trastuzumab loading dose 4 mg/kg on day 1, then 2 mg/kg every week until PD	Anastrozole 1 mg/day until PD	PFS	103	104	28.5	23.9	*p* = 0.325	4.8	2.4	0.63 (0.47, 0.84), *p* = 0.0016
EGF30008	Johnston et al. [17]; Schwartzberg et al. [20]	2009	Postmenopausal women with histologically confirmed stage IIIB/IIIC or IV ER-positive and/or PgR–positive invasive breast cancer; LVEF within the range of normal; ECOG-PS 0–1. No prior therapy for advanced or metastatic disease was allowed	Lapatinib 1500 mg and letrozole 2.5 mg daily until PD	Letrozole 2.5 mg daily with matching lapatinib placebo pill until PD	PFS	111	108	33.3	32.3	0.74 (0.5, 1.1), *p* = 0.113	8.2	3	0.71 (0.53, 0.96), *p* = 0.019
*Chemotherapy A + trastuzumab* versus c*hemotherapy B + trastuzumab*														
Robert 2006	Robert et al. [19]	2006	Women (≥18 years old) with pathologically confirmed, uni- or bidimensionally measurable, HER-2-positive mBC; ECOG-PS 0–2. Patients could not have received prior chemotherapy for mBC	Carboplatin AUC = 6 + paclitaxel 175 mg/m2 every 3 weeks for six cycles trastuzumab 4 mg/kg loading dose, then 2 mg/kg weekly until PD	Paclitaxel 175 mg/m2 every 3 weeks for six cycles + trastuzumab 4 mg/kg loading dose, then 2 mg/kg weekly until PD	ORR	98	98	35.7	32.2	0.9 (0.88, 0.92), *p* = 0.76	10.7	7.1	0.66 (0.59, 0.73), *p* = 0.03
HERNATA Study	Andersson et al. [12]	2010	Women (18 to 75 years old) with HER2-positive mBC or LABC; ECOG-PS ≤ 2; normal LVEF. Prior chemotherapy and HER2-targeted treatment was not allowed for treatment of metastatic or locally advanced disease	Vinorelbine 30 or 35 mg/m2 on days 1 and 8 every 3 weeks until PD + trastuzumab 8 mg/kg loading dose, then 6 mg/kg every 3 weeks until PD	Docetaxel 100 mg/m2 every 3 weeks until PD + trastuzumab 8 mg/kg loading dose, then 6 mg/kg every 3 weeks until PD	TTP	141	143	38.8	35.7	1.01 (0.71, 1.42), *p* = 0.98	15.3	12.4	0.94 (0.71, 1.25), *p* = 0.67
BCIRG 007 Study	Valero et al. [22]	2010	Women (18 to 75 years old) with HER2-amplified mBC, either measurable lesions (RECIST criteria) or nonmeasurable disease including at least two radiologically evident lytic bone lesions, and a Karnofsky performance status ≥60 %. Patients were not eligible if they had received prior platinum salt therapy, chemotherapy, or trastuzumab for mBC	Carboplatin AUC = 6 every 3 weeks for eight cycles + docetaxel 75 mg/m2 weekly every 3 weeks for eight cycles + trastuzumab 4 mg/kg loading dose, then 2 mg/kg on days 1,8, and 15 every 3 weeks for eight cycles, then 6 mg/kg every 3 weeks until PD	Docetaxel 100 mg/m2 on every 3 weeks for eight cycles + trastuzumab 4 mg/kg loading dose, then 2 mg/kg on days 1,8, and 15 every 3 weeks for eight cycles, then 6 mg/kg every 3 weeks until PD	TTP	132	131	37.4	37.1	*p* = 0.99	10.4	11.1	0.914 (0.694, 1.203), *p* = 0.57
NCT00294996	Baselga et al. [14]	2014	Women with HER2-overexpressing mBC and no prior chemotherapy for metastatic disease	NPLD (50 mg/m2 every 3 weeks for six cycles) + trastuzumab (4 mg/kg loading dose followed by 2 mg/kg weekly) + paclitaxel (80 mg/m2 weekly)	Trastuzumab (4 mg/kg loading dose followed by 2 mg/kg weekly) + paclitaxel (80 mg/m2 weekly)	PFS	181	182	33.6	28.9	0.79 (0.61, 1.03), *p* = 0.083	16.1	14.5	0.84 (0.65, 1.08), *p* = 0.174
*Chemotherapy + trastuzumab and pertuzumab*														
CLEOPATRA study	Baselga et al. [13]; Swain et al. [2]; Swain et al. [21]	2013	Women (≥18 years old) with HER2-positive mBC (measurable disease or nonmeasurable disease); LEVF ≥ 50 %; ECOG-PS 0–1. Previous chemotherapy or biological treatment for metastatic disease was not allowed	Pertuzumab 840 mg loading dose, then 420 mg every 3 weeks until PD + trastuzumab 8 mg/kg loading dose, then 6 mg/kg every 3 weeks until PD + docetaxel 75 mg/m2 every 3 weeks for six cycles	Placebo 840 mg loading dose, then 420 mg every 3 weeks until PD + trastuzumab 8 mg/kg loading dose, then 6 mg/kg every 3 weeks until PD + docetaxel 75 mg/m2 every 3 weeks for six cycles	PFS	402	406	56.5	40.8	0.68 (0.56, 0.84), *p* < 0.001	18.7	12.4	0.68 (0.58, 0.80), *p* = 0.001
*Everolimus in trastuzumab-resistant patients*														
BOLERO-1	Hurwitz et al. [32]	2015	Women (≥18 years old) with locally assessed HER2-positive, locally recurrent invasive breast cancer unamenable to resection with curative intent or metastatic disease, with ECOG-PS 0–1, with measurable disease as per RECIST or bone lesions in the absence of measurable disease; no previous systemic therapy for advanced disease was allowed	Everolimus (10 mg/day) + trastuzumab (4 mg/kg loading dose on day 1 with subsequent weekly doses of 2 mg/kg of each 4-week cycle) + paclitaxel (80 mg/m2 on days 1, 8, and 15 of each 4-week cycle)	Placebo + trastuzumab (4 mg/kg loading dose on day 1 with subsequent weekly doses of 2 mg/kg of each 4-week cycle) + paclitaxel (80 mg/m2 on days 1, 8, and 15 of each 4-week cycle)	PFS^a^	480	239	NA	NA	NA	14.95	14.49	0.89 (0.73, 1.08), *p* = 0.1166

*AUC* area under the curve, *CI* confidence interval, *ECOG-PS* Eastern Cooperative Oncology Group performance status, *ER* estrogen receptor*, HER2* human epidermal growth factor receptor 2, *HR* hazard ratio, *LACB* locally advanced breast cancer, *LVEF* left ventricular ejection fraction, *mBC* metastatic breast cancer, *NA* not available, *NPLD* nonpegylated liposomal doxorubicin, *ORR* overall response rate, *OS* overall survival, *PD* progression of disease, *PFS* progression-free survival, *PgR* progesterone receptor,* RECIST* Response Evaluation Criteria In Solid Tumors, *T1* treatment 1, *T2* treatment 2, *TTP* time to progression

^a^Results for the full population, irrespective of the hormone receptor status

#### Chemotherapy ± trastuzumab or lapatinib

The first RCT assessing a therapy against HER2 for metastatic BC that overexpresses HER2 evaluated the efficacy and safety of standard chemotherapy plus trastuzumab versus standard chemotherapy alone (Slamon et al. [1]). In this study, the addition of trastuzumab to chemotherapy was associated with longer OS (25.1 vs. 20.3 months, *p* = 0.046) and longer PFS (6.9 vs. 4.5 months, *p* < 0.001) (Slamon et al. [1]). Inoue and colleagues evaluated the efficacy of sequential therapy with trastuzumab monotherapy followed by trastuzumab plus docetaxel after disease progression versus upfront combination therapy with trastuzumab and docetaxel as first-line therapy in patients with HER2+ metastatic BC. OS and PFS were significantly longer in the upfront trastuzumab and docetaxel group (median values not reported for OS, *p* = 0.04; median PFS of 14.6 vs. 3.7 months, *p* < 0.01) (Inoue et al. [16]). A trial evaluating the efficacy and safety of lapatinib plus paclitaxel compared to placebo plus paclitaxel in patients with newly diagnosed HER2+ metastatic BC showed that the addition of lapatinib to paclitaxel significantly improved OS and PFS versus paclitaxel (27.8 vs. 20.5 months, *p* = 0.0124; 9.7 vs. 6.5 months, *p* <0.001; respectively) (Guan et al. [15]).

#### Chemotherapy + lapatinib versus chemotherapy + trastuzumab

The MA.31 trial assessed the efficacy of lapatinib versus trastuzumab combined with taxanes as first-line treatments for HER2+ metastatic BC over 24 weeks, followed by the same anti-HER2 monotherapy until progression (Gelmon et al. [31]). Median OS was not observed. Median PFS was 9.0 months with lapatinib and 11.3 months with trastuzumab (*p* = 0.001) (Gelmon et al. [31]).

#### Hormone therapy ± trastuzumab or lapatinib

The TAnDEM study was the first phase III RCT combining a hormonal agent and trastuzumab without chemotherapy as treatment for HER2/hormone receptor–copositive metastatic BC (Kaufman et al. [18]). Postmenopausal women were randomly assigned to anastrozole with or without trastuzumab until progression; those receiving the monoclonal antibody in addition to anastrozole experienced a non-statistically significant improvement in OS (28.5 vs. 23.9 months, *p* = 0.325), but a statistically significant longer PFS (4.8 vs. 2.4 months, *p* = 0.0016) (Kaufman et al. [18]). A further study evaluated the effect of adding lapatinib, a dual tyrosine kinase inhibitor blocking both the epidermal growth factor receptor and the HER2 receptor, to the aromatase inhibitor letrozole in women with hormone receptor-positive metastatic BC (Johnston et al. [17]). The OS and the PFS in the HER2+ population were of 33.3 months and 8.2 months, respectively, in patients receiving lapatinib plus letrozole and 32.3 months and 3.0 months, respectively, in patients receiving letrozole (*p* = 0.113 and *p* = 0.019, respectively) (Johnston et al. [17]).

#### Chemotherapy A + trastuzumab versus chemotherapy B + trastuzumab

In 2006, the result of a trial evaluating the clinical benefit and safety of the addition of carboplatin to trastuzumab and paclitaxel versus trastuzumab and paclitaxel alone, reported no difference in OS (35.7 vs. 32.2 months, *p* = 0.76), but a significant improvement in PFS (10.7 vs. 7.1 months, *p* = 0.03) with the inclusion of carboplatin (Robert et al. [19]). The HERNATA study was designed to compare docetaxel with vinorelbine, both associated with trastuzumab, as first-line therapy of HER2+ locally advanced or metastatic BC (Andersson et al. [12]). In that trial, the OS and the PFS were of 38.8 months and 15.3 months, respectively, in the vinorelbine plus trastuzumab group and 35.7 months and 12.4 months, respectively, in the docetaxel plus trastuzumab group (*p* = 0.98 and *p* = 0.67) (Andersson et al. [12]). In the BCIRG 007 study, patients were randomly assigned to trastuzumab plus docetaxel or trastuzumab plus carboplatin and docetaxel, but no statistically significant differences were found between groups in OS (37.1 and 37.4 months, respectively; *p* = 0.99) and PFS (10.4 vs. 11.1 months, *p* = 0.57) (Valero et al. [22]).

Since the addition of trastuzumab to anthracyclines resulted in high incidence of cardiac toxicity in the pivotal trial conducted by Slamon and colleagues, Baselga and colleagues conducted a trial of first-line nonpegylated liposomal doxorubicin plus trastuzumab and paclitaxel versus trastuzumab and paclitaxel alone in patients with HER2+ metastatic BC (Slamon et al. [1]; Baselga et al. [14]). The OS was 33.6 and 28.9 months, respectively (*p* = 0.083), and the median PFS was 16.1 and 14.5 months, respectively (*p* = 0.174) (Baselga et al. [14]). Thus, this trial failed to demonstrate a significant clinical improvement with the addition of nonpegylated liposomal doxorubicin to the trastuzumab and paclitaxel regimen (Baselga et al. [14]).

#### Chemotherapy + trastuzumab and pertuzumab

In the CLEOPATRA study, patients with HER2+ metastatic BC who had not received previous chemotherapy or biological treatment for their metastatic disease were assigned to receive pertuzumab plus trastuzumab and docetaxel or placebo plus trastuzumab and docetaxel (Swain et al. [21]). Median OS in the pertuzumab group was estimated to be 56.5 months, compared to 37.6 months in the placebo (*p* < 0.001); the PFS was 18.7 in the pertuzumab group versus 12.4 months in the placebo group (*p* < 0.001) (Swain et al. [2]; Swain et al. [21]).

#### Everolimus in trastuzumab-resistant patients

The BOLERO-1 trial included women with HER2+, trastuzumab-resistant, locally recurrent invasive BC unamenable to resection with curative intent or metastatic BC who had not received previous trastuzumab or chemotherapy for the advanced stage of the disease (Hurvitz et al. [32]). Patients were randomly assigned to daily everolimus or placebo plus weekly trastuzumab and paclitaxel. OS analyses are still in progress. The median PFS was 14.95 months with everolimus and 14.49 months with placebo (*p* = 0.1166) in the full population; 20.27 months with everolimus and 13.08 with placebo in the hormone receptor-negative population (*p* = 0.0049; did not cross the protocol-specified threshold of *p* = 0.0044) (Hurvitz et al. [32]).

### Second-line metastatic BC and beyond

**Fig. 3:**
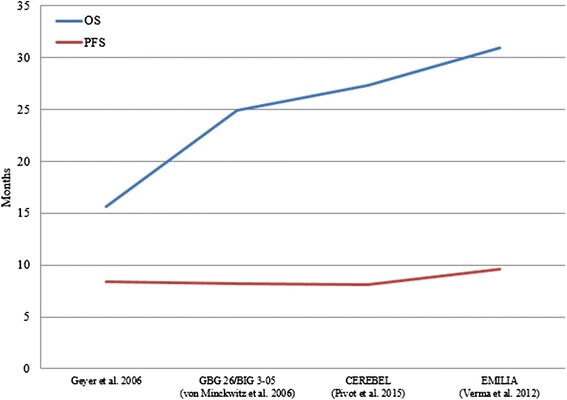
Overall survival (*blue*) and progression-free survival (*red*) of patients diagnosed with metastatic breast cancer receiving second-line treatment, according to the most effective treatment in each study. Results are displayed from the study with lower OS to the study with higher OS. Results from TH3RESA and BOLERO-3 are not displayed because OS results were not provided. GBG 26/BIG 03–05, German Breast Group 26/Breast International Group 03–05 study. *OS* overall survival, *PFS* progression-free survival

Seven phase III RCTs (11 publications) evaluated therapies in the second-line setting or beyond (Table 2) (André et al. [23]; Blackwell et al. [24]; Blackwell et al. [5]; Cameron et al. [3]; Cameron et al. [25]; Geyer et al. [26]; Krop et al. [27]; Pivot et al. [28]; Verma et al. [4]; von Minckwitz et al. [29]; von Minckwitz et al. [30]). Figure 3 presents the OS (*blue*) and PFS (*red*) results for patients diagnosed with metastatic BC receiving second-line or beyond treatment, according to the most effective treatment in each study.

**Table 2:** Summary of design and results of studies assessing second-line or beyond therapies for the treatment of patients diagnosed with HER2-positive metastatic breast cancer

Clinical trial	Reference	Year	Target population	T1	T2	Primary endpoint of efficacy	Patients on T1, *n*	Patients on T2, *n*	OS T1	OS T2	HR (95 % CI), *p* value	PFS T1	PFS T2	HR (95 % CI), *p* value
*Chemotherapy ± trastuzumab or lapatinib*														
Geyer 2006	Geyer et al. [26]; Cameron et al. [3]; Cameron et al. [25]	2006	Women with progressive, HER2-positive, locally advanced or metastatic breast cancer who had previously been treated with a minimum of an anthracycline, a taxane and trastuzumab	Lapatinib 1250 mg daily + capecitabine at a dose of 2000 mg/m2 in two divided doses on days 1 through 14 of a 21-day cycle	Capecitabine 2500 mg/m2 in two divided doses on days 1 through 14 of a 21-day cycle	TTP	198	201	15.6	15.3	0.78 (0.55, 1.12) *p* = 0.177	8.4	4.1	0.47 (0.33, 0.67), *p* < 0.001
A German Breast Group 26/Breast International Group 03–05 study	von Minckwitz et al. [29]; von Minckwitz et al. [30]	2009	Women with pathologically confirmed, HER-2–positive, locally advanced or metastatic breast cancer	Capecitabine 2500 mg/m2 (1250 mg/m2 twice daily) on days 1 through 14 followed by 1 week of rest	Capecitabine 2500 mg/m2 (1250 mg/m2 twice daily) on days 1 through 14 followed by 1 week of rest + trastuzumab 6 mg/kg body weight as a 30-minute infusion every 3 weeks until PD	TTP	78	78	20.6	24.9	0.76 (0.48, 1.22) *p* = 0.257	5.6	8.2	0.69 (0.48, 0.97), *p* = 0.034
*Chemotherapy + trastuzumab or chemotherapy + lapatinib*														
CEREBEL^a^	Pivot et al. [28]	2015	Women with HER2-positive mBC and without baseline CNS metastases. Patients were required to have received prior anthracycline and/or taxanes for (neo)adjuvant or metastatic disease. Prior trastuzumab was allowed but not required	Trastuzumab infusion of 6 mg/kg every 3 weeks (with possibly a loading dose of 8 mg/kg on day 1) and capecitabine 2500 mg/m2 per day on days 1 through 14, every 21 days	Lapatinib 1250 mg once daily and capecitabine 2000 mg/m2 per day on days 1 through 14, every 21 days	Incidence of CNS metastases as first site of relapse	269	271	27.3	22.7	1.34 (0.95, 1.64), *p* = 0.095	8.1	6.6	1.30 (1.04, 1.64), *p* = 0.021
*Lapatinib + trastuzumab*														
EGF104900 study	Blackwell et al. [24]; Blackwell et al. [5]	2012	Women with ErbB2-positive mBC who experienced progression on prior trastuzumab-containing regimens	Lapatinib 1000 mg daily in combination with intravenous trastuzumab 2 mg/kg weekly (after the initial 4 mg/kg loading dose)	Lapatinib 1500 mg daily	PFS	148	148	12.04	9.1	0.75 (0.53, 1.07) *p* = 0.106	2.8	1.9	0.73 (0.57, 0.93), *p* = 0.008
*Trastuzumab emtansine (T-DM1)*														
EMILIA	Verma et al. [4]	2012	HER2-positive advanced breast cancer previously treated with trastuzumab and a taxane	T-DM1 3.6 mg/kg every 3 weeks until PD	Lapatinib 1250 mg/day + capecitabine 1000 mg/m2 twice a day on days 1–14 for 3 weeks until PD	PFS, OS	495	496	30.9	25.1	0.68 (0.55, 0.85) *p* < 0.001	9.6	6.4	0.65 (0.55, 0.77), *p* < 0.001
TH3RESA	Krop et al. [27]	2014	Women (≥18 years, LVEF ≥ 50 %, ECOG-PS 0–2) with progressive HER2-positive advanced breast cancer who had received two or more HER2-directed regimens in the advanced setting, including trastuzumab and lapatinib, and previous taxane therapy in any setting	Trastuzumab emtansine (3.6 mg/kg intravenously every 21 days)	Physician’s choice	PFS, OS	404	198	NYR	14.9	0.552 (0.369, 0.826), *p* = 0.0034	6.2	3.3	0.528 (0.422, 0.661), *p* < 0.0001
*Everolimus in trastuzumab-resistant patients*														
BOLERO-3	André et al. [23]	2014	Women with HER2-positive, trastuzumab-resistant, advanced breast carcinoma who had previously received taxane therapy	Daily everolimus (5 mg/day) plus weekly trastuzumab (2 mg/kg) and vinorelbine (25 mg/m(2))	Placebo plus trastuzumab plus vinorelbine, in 3-week cycles	PFS	284	285	NA	NA	NA	7.00	5.78	0.78 (0.65, 0.95), *p* = 0.0067

*CI* confidence interval, *CNS* central nervous system, *ECOG-PS* Eastern Cooperative Oncology Group performance status, *HER2* human epidermal growth factor receptor 2, *HR* hazard ratio, *LVEF* left ventricular ejection fraction *mBC* metastatic breast cancer, *NA* not available, *NYR* not yet reached, *OS* overall survival, *PD* progression of disease, *PFS* progression-free survival, *T1* treatment 1, *T2* treatment 2, *T-DM*1 trastuzumab emtansine,*TTP* time to progression

^a^CEREBEL was included in the list of studies assessing second-line or beyond therapies because patients were required to have received prior chemotherapy for (neo)adjuvant or metastatic disease, according to the inclusion criteria of the study. However, it should be noted that nearly 43 % of patients in the lapatinib plus capecitabine arm (117 of 271 patients) and 45 % of patients in the trastuzumab plus capecitabine arm (121 of 269 patients) had not received prior treatment for the metastatic disease

#### Chemotherapy ± trastuzumab or lapatinib

The study by Geyer et al. [26], which included women with HER2+, locally advanced or metastatic BC that had progressed after trastuzumab-based therapy, who were randomly assigned to receive lapatinib plus capecitabine or capecitabine alone, did not report results on OS (Geyer et al. [26]), but an updated efficacy analysis showed that the OS was 15.6 months in patients receiving lapatinib plus capecitabine and 15.3 months in the capecitabine alone group (*p* = 0.177) (Cameron et al. [3]); while time to progression was of 8.4 months and 4.1 months, respectively (*p* < 0.001).

The GBG 26/BIG 3–05 phase III study, which included patients with HER2+ locally advanced or metastatic BC who had progressed during treatment with trastuzumab, reported no improvement in OS, but a statistically significant improvement of PFS in patients that received capecitabine with continuation of trastuzumab compared with those that received capecitabine alone (24.9 vs. 20.6 months, *p* = 0.73; 8.2 vs. 5.6 months, *p* = 0.0338, respectively) (von Minckwitz et al. [29], von Minckwitz et al. [30]). This trial changed an oncology paradigm since it showed that continuing trastuzumab beyond progression (associated with a different anticancer agent) was beneficial (von Minckwitz et al. [29], von Minckwitz et al. [30]).

#### Chemotherapy + trastuzumab or chemotherapy + lapatinib

Female patients with HER2+ metastatic BC and without central nervous system metastases were randomly assigned to receive lapatinib plus capecitabine or trastuzumab plus capecitabine in the CEREBEL trial (Pivot et al. [28]). Patients were required to have received prior chemotherapy for (neo)adjuvant or metastatic disease. Nevertheless, approximately 43 % of patients in the lapatinib plus capecitabine arm and 45 % of patients in the trastuzumab plus capecitabine arm had not received prior treatment for the metastatic disease (Pivot et al. [28]). PFS was longer with trastuzumab plus capecitabine (8.1 vs. 6.6 months, *p* = 0.021), with no differences in OS (27.3 vs. 22.7 months, *p* = 0.095) (Pivot et al. [28]).

#### Lapatinib + trastuzumab or lapatinib monotherapy

In the EGF104900 study, patients with heavily pretreated HER2+ metastatic BC were randomly assigned to receive lapatinib plus trastuzumab or lapatinib monotherapy as a third-line anti-HER treatment (Blackwell et al. [5]). Combination lapatinib plus trastuzumab significantly improved OS and PFS (14 vs. 9.5 months, *p* = 0.026; and 2.6 vs. 1.9 months, *p* = 0.010; respectively) (Blackwell et al. [5]).

#### Trastuzumab emtansine (T-DM1)

Lapatanib plus capecitabine were further compared with trastuzumab emtansine (T-DM1) in the EMILIA trial that showed a statistically significant improvement of OS and PFS in patients treated with the latter regimen (25.1 vs. 30.9 months and 9.6 vs. 6.4 months, respectively, with *p* < 0.001 for both end points) (Verma et al. [4]).

In the TH3RESA trial patients with HER2+ unresectable locally advanced or recurrent BC or metastatic BC who had been treated with two or more HER2-directed regimens, including trastuzumab and lapatinib, were randomized to receive T-DM1 or treatment of physician’s choice (Krop et al. [27]). A trend favoring T-DM1 was observed on OS (*p* = 0.0034), but the stopping boundary was not crossed. PFS was significantly improved with T-DM1 (median 6.2 months vs. 3.3 months; *p* < 0.001) (Krop et al. [27]).

#### Everolimus in trastuzumab-resistant patients

The BOLERO-3 trial included women with HER2+, trastuzumab-resistant, advanced BC who had previously received taxane therapy (André et al. [23]). Patients were randomly assigned to daily everolimus plus weekly trastuzumab and vinorelbine or to placebo plus trastuzumab and vinorelbine. OS was not mature and median PFS was 7 months with everolimus and 5.8 months with placebo (*p* = 0.0067) (André et al. [23]).

## Discussion and conclusions

The advances in early diagnosis and adjuvant pharmacological therapy have led to a decrease in mortality rates from BC in developed countries (Cardoso et al. [8]). Nonetheless, the prevalence of metastatic BC is still high. Many women live with the disease for several years, but there is, however, a lack of accurate data for this condition since most cancer registries do not capture relapses (Cardoso et al. [8]). Metastatic BC is not considered curable and the treatment goal includes improving patients’ quality of life and prolonging survival as much as possible (Cardoso et al. [8]).

In the early 1990s the median OS of patients diagnosed with metastatic BC was only 14 months (Chia et al. [33]). The introduction of more efficacious therapies has substantially improved the prognosis of these patients. The addition of HER2-targeted therapies to standard treatments is associated with marked improvement of OS in patients with metastatic BC. The first approved HER2-directed inhibitor, trastuzumab, led to an OS prolongation of 5 months when added to chemotherapy (Slamon et al. [1]), and became the standard of care in first-line treatment (Cardoso et al. [8]). The addition of lapatinib to paclitaxel also results in a prolongation of 7 months of OS compared with paclitaxel alone, as well as the prolongation of PFS (Guan et al. [15]). More recently, the MA.31 trial provided an answer to a relevant clinical question through a head-to-head comparison between trastuzumab and lapatinib (both combined with taxanes) as first-line treatments for HER2+ metastatic breast cancer (Gelmon et al. [31]). Compared to trastuzumab, lapatinib was associated with a shorter PFS and a worse safety profile, with increased diarrhea, appetite loss, social functioning, and skin rash (Gelmon et al. [31]). These results support the use of trastuzumab over lapatinib in patients with treatment-naïve HER2+ metastatic breast cancer, and have implications for clinical practice (Gelmon et al. [31]).

Other therapeutic combinations were evaluated in clinical trials, but not all led to the prolongation of patients’ survival. The addition of trastuzumab to the hormonal agent, anastrozole, was not associated with an increase of OS, despite an increase of 2.4 months in PFS, when compared with anastrazole monotherapy (Kaufman et al. [18]). The lapatinib plus letrozole combination increased PFS by 5 months when compared with letrozole monotherapy, but both regimens had similar OS (approximately 33 months) (Johnston et al. [17]). Adding carboplatin to trastuzumab and taxane did not extend OS and was associated with increased rates of neutropenia and thrombocytopenia (Robert et al. [19]; Valero et al. [22]). The BOLERO-1 trial showed that the addition of everolimus to trastuzumab and paclitaxel did not result in an improvement of PFS in patients with HER2+ advanced breast cancer (Hurwitz et al. [32]); a clinically important prolongation of PFS (7.2 months) was only seen with everolimus (versus placebo) when the analysis was restricted to hormone receptor-negative patients, but even so the *p* value did not achieved the prespecified criteria for statistical significance (Hurwitz et al. [32]). Further, everolimus was associated with an increased risk for serious adverse events, such as neutropenia, leucopenia, anemia, febrile neutropenia, stomatitis, and fatigue (Hurwitz et al. [32]). In all these studies evaluation of tolerability and quality of life is as relevant as efficacy results.

Dual HER2 inhibition with trastuzumab and pertuzumab, a HER2-targeted humanized monoclonal antibody that inhibits dimerization, was more active than single inhibition with trastuzumab (Swain et al. [2]). This therapeutic combination prolonged patients’ survival, as well as PFS across all predefined subgroups. In the CLEOPATRA study, this led to an unprecedented PFS of 18 months and median OS of almost 5 years (Swain et al. [2]). Caution however is needed when extrapolating these results to current patient populations since only 10 % of patients from this trial had received prior chemotherapy with or without adjuvant or neoadjuvant trastuzumab.

HER2-targeted therapies have also demonstrated efficacy in the treatment of metastatic BC in the second-line setting. When compared to lapatinib plus capecitabine, T-DM1, an antibody-drug conjugate of trastuzumab and the chemotherapy drug DM1 (emtansine), extended the OS for almost 6 months in patients who had previously received trastuzumab and a taxane (Verma et al. [4]). Additionally, patients treated with T-DM1 experienced less toxicity. Similarly, the addition of everolimus to trastuzumab and vinorelbine in patients with trastuzumab-resistant and taxane-pretreated HER2+ advanced BC was associated with an improvement in PFS in this population. However, the addition of everolimus was associated with a high incidence of serious adverse events, exhibiting a similar safety profile to that seen in the BOLERO-1 trial (André et al. [23]). Beyond the second-line setting, a study demonstrated that treatment with dual HER2 blockade lapatinib plus trastuzumab improved OS when compared with lapatinib (Blackwell et al. [5]). However, treatment with lapatinib plus trastuzumab was associated with higher rates of treatment discontinuation and toxicity. More recently, the benefit of T-DM1, mainly on PFS, was also demonstrated in patients with HER2+ metastatic BC who have previously received trastuzumab and lapatinib (Krop et al. [27]).

Patients treated with trastuzumab have higher risk of cardiotoxicity, mainly asymptomatic left ventricular systolic dysfunction or congestive heart failure (Slamon et al. [1]; Seidman et al. [34]). Congestive heart failure was detected as a safety signal late in the development of the drug (Seidman et al. [34]). Yet, the rates of cardiac dysfunction were greatest when trastuzumab was administered concomitantly with anthracyclines, and the most frequent events were asymptomatic declines in left ventricular ejection fraction (Seidman et al. [34]; Morris and Hudis [35]). Evidence suggests that trastuzumab’s cardiotoxicity is, however, uncommon and generally reversible (Morris and Hudis [35]; Procter et al. [36]; de Azambuja et al. [37]). In order to minimize the risk, all candidates for treatment with trastuzumab must undergo a baseline cardiac assessment, as well as regular (every 3 months) assessments during treatment, in particular those who have been previously exposed to anthracyclines or with risk factors for cardiac events. Despite this small cardiac iatrogenic potential, in the majority of patients with HER2+ metastatic BC, the risk of cardiac dysfunction is justified given the improvement in OS associated with trastuzumab (Seidman et al. [34]). Moreover, the most recently developed HER2-targeted therapeutic alternatives have been associated with relatively low rates of cardiovascular events (Verma et al. [4]).

Another safety issue is the, apparently, high rate of central nervous system metastases in patients receiving adjuvant trastuzumab, raising the question whether this treatment could predispose for central nervous system recurrence (Yin et al. [38]). A prospective, observational study of HER2+ metastatic BC patients concluded that the use of trastuzumab, chemotherapy, and surgery following central nervous system metastases were each associated with longer survival (Brufsky et al. [39]). Furthermore, a retrospective study found that anti-HER2 treatment is associated with longer time to occurrence of brain metastases when given before brain metastases diagnosis and with survival benefit when given after brain metastases (Yap et al. [40]). Findings from the CEREBREL clinical trial demonstrated no difference in the incidence of brain metastasis as a first site of progression between lapatinib plus capecitabine and trastuzumab plus capecitabine (Pivot et al. [28]). The most likely explanation for the data is that the beneficial effect of trastuzumab on systemic disease extends the survival of patients to such a degree that central nervous system metastases became clinically evident (Dawood et al. [41]; Yin et al. [38]).

In clinical practice different combinations than those evaluated in clinical trials may be used, assuming that the benefit of trastuzumab is the same regardless of its partner agent (Pegram et al. [42]; Pegram et al. [43]; Harris et al. [44]). Only a small number of HER2 combination therapies have been evaluated in RCTs, as it was noted in a previous meta-analysis (Harris et al. [44]), but it would be practically and economically unfeasible and unrealistic to perform a phase III trial to evaluate all possible combinations. It is now unethical to perform a study evaluating the addition of an anti-HER2 agent to a given cytotoxic or combination of cytotoxic agents versus not, the only design that would indeed assess the value of the different combinations. One can only compare between different combinations. Furthermore, the development of new anti-HER2 agents and the potential of dual or even triple blockade raises other possibly more relevant questions to test. The optimal sequencing of HER2-targeted therapies across multiple lines of treatment is of utmost importance due to the increasing number of available drugs (Verma et al. [4]).

There are several other studies evaluating different combinations of cytotoxic and anti-HER2 agents. However, such studies usually include a limited number of patients and do not report on OS. Therefore we chose to select only phase III RCTs since this design is less susceptible to bias, providing the highest level clinical and statistical evidence (Straus et al. [45]).

HER2-targeted therapies have an undeniable favorable impact in the outcome of patients with HER2+ metastatic BC. Integration of trastuzumab led to about 6 months benefit in OS. More recently, T-DM1 after trastuzumab led to a further 6 months benefit in OS. HER2 dual blockade with pertuzumab and trastuzumab is associated with a survival extension to more than 4.5 years (Swain et al. [2]). Since 2001, comparing the results of the clinical trial of Slamon and colleagues evaluating the addition of trastuzumab to chemotherapy, there has been an increase in OS of 2.5 years with anti-HER2 drugs (Slamon et al. [1]). Survival improvements have also been noted for the second- and third-line therapies. Once a poor prognosis disease, HER2+ advanced BC has now a wide range of therapeutic options that significantly increased the OS of patients and has become the subtype of metastatic BCs with the longest medium survival, similar to luminal breast cancers.

## Additional file

Additional file 1:
**Search strategies used to retrieve randomized controlled trials from the Cochrane Library and PubMed (Updated, 6 October 2015).** (DOCX 12 kb)

## Abbreviations

BC: Breast cancer
HER2+: Human epidermal growth factor receptor 2 positive
ITT: intention-to-treat
mBC: Metastatic breast cancer
OS: Overall survival
PFS: Progression-free survival
RCTs: Randomized controlled trials
T-DM1: Trastuzumab emtansine
