# Supplementary material for: The benefit of HER2-targeted therapies on overall survival of patients with metastatic HER2-positive breast cancer – a systematic review
Source: Breast Cancer Res. 2015 Nov 17;17:140. doi: 10.1186/s13058-015-0648-2 (PMC4650834; doi:10.1186/s13058-015-0648-2)
Supplement: Additional file 1: — Search strategies used to retrieve randomized controlled trials from the Cochrane Library and PubMed (Updated, 6 October 2015). (DOCX 12 kb) [file 13058_2015_648_MOESM1_ESM.docx]

Appendix – Search strategies used to retrieve Randomized Controlled Trials from the Cochrane Library and Pubmed (Updated, 06-10-2015).

**COCHRANE LIBRARY**

| **N** | **Search strategy** | **Results** |
| --- | --- | --- |
| #1 | (("advanced" OR "metastatic") AND ("breast cancer")) AND ("HER2" OR "HER-2" OR "Human epidermal growth factor receptor-2-positive" OR “erbB-2” OR “erbB2” OR “erbB 2”) | 780 |
| #2 | "Randomized Controlled Trial" OR "Randomized Controlled Trials as Topic" | 457408 |
| #3 | #1 AND #2 | 301 |
| #4 | Filter: Trials | 267 |

**PUBMED**

| **N** | **Search strategy** | **Results** |
| --- | --- | --- |
| #1 | (("advanced" OR "metastatic") AND ("breast cancer")) AND ("HER2" OR "HER-2" OR "Human epidermal growth factor receptor-2-positive" OR “erbB-2” OR “erbB2” OR “erbB 2”) | 4658 |
| #2 | "Randomized Controlled Trials as Topic"[Mesh] OR "Randomized Controlled Trial" [Publication Type] | 490128 |
| #3 | #1 AND #2 | 367 |
